# Supplementary material for: Electromagnetic Noise Characterization and Suppression in Low‐Field MRI Systems
Source: Magn Reson Med. 2026 Jan 16;95(5):3000–7. doi: 10.1002/mrm.70235 (PMC12962213; doi:10.1002/mrm.70235)
Supplement: Supplementary file 1 — Data S1: mrm70235‐sup‐0001‐Supinfo.pdf. [file MRM-95-3000-s001.pdf]

Received XXX; | Revised XXX; | Accepted XXX
DOI: xxx/xxxx

NOTE

Supporting Information - Electromagnetic Noise Characterization and Suppression in Low-Field MRI Systems

Teresa Guallart-Naval | José M. Algarín | Joseba Alonso

[1]MRILab, Institute for Molecular Imaging and Instrumentation (i3M), Spanish National Research Council (CSIC) and Universitat Politècnica de València (UPV), 46022 Valencia, Spain

Correspondence
Joseba Alonso. Email: joseba.alonso@i3m.upv.es

Wiring and Grounding Recommendations

Clean, carefully planned wiring and grounding are absolutely essential in low-field MRI systems. Electromagnetic interference (EMI) often enters the receive chain through flawed cable practices, poorly shielded electronics, or improper ground referencing. The following sections provide detailed and experience-based guidance to help avoid these pitfalls.

1 | GENERAL CABLING

- Cables are often **the** problem — and often an elusive one. Noise issues that seem random or untraceable frequently originate from overlooked cable faults.
- Handle cables with extreme care: do not stretch, twist, or kink them. Use strain reliefs and avoid placing them where they may be stepped on or pinched. RF cables (coaxial) are particularly delicate and should be protected at all times.
- Use high-quality cables from reliable vendors.
- Label and color-code all cables for clarity, maintenance, and debugging.
- Keep cables as short and thick as possible. This is especially critical for power, ground, and gradient cables, where low resistance reduces voltage drops and power dissipation.
- Minimize the number of cable transitions and interconnections. Each connector introduces impedance discontinuities and potential noise entry points.
- Avoid coiling cables, which creates inductive loops prone to antenna effects and ground loops.

2 | RF CABLES

- Long coaxial cables can lead to resonant cavity effects at low-field RF wavelengths. Keep them short and use as few transitions as possible.
- Never use T-pieces. If monitoring or tapping is required, use directional couplers or RF power splitters.
- When RF cables connect to a Faraday enclosure, ensure a solid electrical connection between the cable connector and the metal casing.
- Consider using cable traps or baluns to suppress common-mode currents that may flow along the outer shield of coaxial cables.

3 | NON-RF CABLES

- These cables are often unshielded and can pick up or radiate EMI. Use shielding sleeves and ensure

proper grounding of power, gradient, and digital cables (see Sec. 5).

- Again, cable traps and baluns may be useful for suppressing unwanted currents.
- Be especially careful with gradient cables — they are a major source of noise in low-field systems.

4 | METALLIC ENCLOSURES

- Enclose all electronics in conductive casings (e.g., aluminum boxes). While commercial RF modules are usually well-shielded, homemade circuits (e.g., TxRx switches, LNAs, tuning/matching units) require close attention.
- Ensure strong electrical contact between all parts of the enclosure (e.g., screw down lids firmly, ensure contacts are through large surfaces, rather than point contacts).
- Ensure low-impedance ground contact between printed circuit boards and the inside of the containing boxes.
- Avoid unshielded openings or through-hole connections. Use proper panel connectors wherever possible.

5 | SCANNER SHIELDINGS

5.1 | Internal shielding

- The system's ground reference is defined by the return line of the RF coil. To isolate this from gradient-induced noise, an inner shield is typically used — often a cylindrical copper sleeve.
- Ensure a robust connection (short and thick) between the coil return and the inner shield, ideally soldered or screwed at both ends.
- Inner shields are thin and prone to mechanical degradation. If noise suddenly increases, they should be among the first suspects.
- While thicker shields provide better RF isolation, they may introduce eddy current issues if not properly slotted or segmented[1].

5.2 | External shielding

- Ideally, the external shield fully encloses the scanner core (magnet and gradient system), leaving only the bore openings.
- If the shield is assembled from multiple parts (e.g., barrel, lids, baseplate), ensure robust electrical contact at all joints.
- Ensure a high-quality connection between internal and external shields. This allows the more mechanically robust external shield to serve as a grounding point for cable sleeves, Faraday cages, and other subsystems. Follow a star grounding pattern wherever possible[2].

6 | NOISE SOURCES

- Keep all "dirty" components as far as possible from the RF chain and gradient cables. This includes digital electronics, control computers, power supplies, and switching regulators.
- Route "dirty" cables (digital, high-power, etc.) away from the RF path and gradient cables.
- Avoid switch-mode power supplies whenever possible. Linear power supplies are strongly preferred, but should be placed far enough from the magnet to avoid B_0 50 or 60 Hz modulation if they contain magnetic cores in the transformer.

7 | DOCUMENTATION

- Maintain a detailed grounding and connection diagram, including as many system elements as possible — ideally all of them. Update the diagram whenever any change is made.
- Keep logs of all tests, results, and relevant observations. Fighting noise often involves trial, error, and time-dependent behavior. Historical notes are often invaluable when diagnosing persistent or recurring issues.

ORCID

Teresa Guallart-Naval 0000-0002-6617-9563
José M. Algarín 0000-0003-2644-7225
Joseba Alonso 0000-0002-2721-1380

REFERENCES

1. B. de Vos, R. Remis, and A. Webb, “Segmented RF shield design to minimize eddy currents for low-field Halbach MRI systems,” Journal of Magnetic Resonance, vol. 362, p. 107669, 5 2024.
2. E. B. Joffe and K.-S. Lock, Grounds for grounding: A circuit to system handbook. John Wiley & Sons, 2011.

□
